# Global perspective on gaps in fungal diagnostics in low-resource settings: WHO landscape analysis and research priorities for invasive fungal diseases

**DOI:** 10.1016/j.lanmic.2025.101307

**Published:** 2026-05

**Authors:** Maurine Murtagh, P Lewis White, Juan Luis Rodriguez-Tudela, Ana Alastruey-Izquierdo, Sharon C-A Chen, Philippe J Dufresne, Beatriz L Gómez, Guillermo Garcia-Effron, Betsy Wonderly Trainor, Pilar Garcia-Vello, Till T Bachmann, Pascale Ondoa, Daniel Marcano Zamora, Tamarie Rocke, Alexandra Cameron, Valeria Gigante

**Affiliations:** a The Murtagh Group, Woodside, CA, USA; b Antimicrobial Resistance (AMR) Department, WHO, Geneva, Switzerland; c Public Health Wales Microbiology, Cardiff, UK; d Global Action for Fungal Infections, Geneva, Switzerland; e Mycology Reference Laboratory, National Centre for Microbiology, Instituto de Salud Carlos III, Madrid, Spain; f Clinical Mycology Reference Laboratory, Centre for Infectious Diseases and Microbiology Laboratory Services, Institute of Clinical Pathology and Medical Research, Westmead Hospital, Westmead, NSW, Australia; g Mycology Reference Laboratory, Laboratoire de Santé Publique du Québec, Institut National de Santé Publique du Québec, Sainte-Anne-de-Bellevue, QC, Canada; h Translational Microbiology and Emerging Diseases Study Group (MICROS), School of Medicine and Health Sciences, Universidad del Rosario, Bogota, Colombia; i Laboratorio de Micología y Diagnóstico Molecular, Facultad de Bioquímica y Ciencias Biológicas, Universidad Nacional de Litoral, Instituto de Salud y Ambiente Consejo Nacional de Investigaciones Científicas y Técnicas (CONICET), Santa Fe, Argentina; j Combating Antibiotic-Resistant Bacteria Biopharmaceutical Accelerator (CARB-X), Boston, MA, USA; k Centre for Inflammation Research Institute for Regeneration and Repair, University of Edinburgh, Edinburgh, UK; l The Global Fund to Fight AIDS, Tuberculosis and Malaria, Geneva, Switzerland; m Amsterdam Institute for Global Health and Development, Department of Global Health, University of Amsterdam, Amsterdam, Netherlands

## Abstract

Invasive fungal diseases (IFDs) are a growing global health threat, particularly in low-income and middle-income countries (LMICs), where diagnostic capacity is limited. The emergence and spread of antifungal resistance further complicate clinical management. Despite the high burden of IFDs, diagnostic development for fungal pathogens has lagged compared with that for its bacterial and viral counterparts. In this Review, we synthesised findings from WHO’s 2024 diagnostic landscape analysis of fungal priority pathogens. We discussed the availability, accessibility, and performance of the current diagnostic tools, including phenotypic, immunological, and molecular methods, and identified key gaps in LMICs. WHO’s research and development priorities for diagnostics for LMICs are also presented herein. The assessment reveals that most diagnostic platforms for IFDs are culture dependent, infrastructure intensive, and inaccessible at primary and secondary health-care levels in LMICs. Non-culture-based methods, such as lateral flow immunoassays and molecular diagnostics, are promising, but remain restricted in scope, species coverage, and affordability. Multiplex platforms capable of simultaneous broad pathogen detection and antifungal resistance testing are scarce. WHO has identified priority areas for diagnostic innovation, including simplified lateral flow immunoassays, automated culture systems, and integrated molecular platforms suitable for use in LMICs. Addressing diagnostic gaps for IFDs requires targeted investment in simplified, rapid, and affordable diagnostic tools that can be deployed across multiple levels of health-care system. WHO’s research and development priorities aim to guide diagnostic developers and public health stakeholders in accelerating innovation and improving access to fungal diagnostics, particularly in LMICs, to reduce IFD-related morbidity and mortality.

## Introduction

Invasive fungal diseases (IFDs) pose a growing global health burden, particularly affecting individuals with compromised immunity and clinically vulnerable populations.^1^, ^2^, ^3^ Groups at high risk include individuals with cancer, HIV/AIDS, primary immunodeficiencies, and chronic lung disease, and recipients of solid organ or stem-cell transplants. Critically ill patients in intensive care, especially those on mechanical ventilation or with severe respiratory infections, are also highly susceptible.^2^^,^^4^ Other at-risk populations include neonates, older individuals (aged 65 years and older), malnourished or displaced individuals, and those injured by conflict or natural disasters.^5^ Endemic systemic mycoses (eg, histoplasmosis, coccidioidomycosis, blastomycosis, and talaromycosis) disproportionately affect individuals in specific geographical regions due to environmental exposure.^6^ Climate change is expanding the range of endemic fungi, enabling the emergence of new pathogens and contributing to antifungal resistance (AFR).^7^

**Search strategy and selection criteria**

The methodology for the Review included three components: desktop review focusing on publicly available data, literature review, and expert consultations.

The desktop review consisted of an extensive review of relevant available information, including, but not restricted to published reports from recognised health organisations (eg, Unitaid, Foundation for Innovative New Diagnostics [FIND], WHO, and others), conference reports, abstracts, and posters, as well as WHO reports, systematic reviews, and discussions with WHO staff and external experts. For individual commercial diagnostic platforms, company websites, US Food and Drug Administration website, European Medicines Agency website, and websites of other stringent regulatory authorities were searched to derive operational characteristics of the profiled devices or tests as supplied by the companies and developers or publicly available information, or both.

For the literature review, Embase, Cochrane Database of Systematic Reviews, and PubMed, among others, were searched from Jan 1, 2024, to July 31, 2024. The search terms included combinations of terms such as “in vitro diagnostics”, “invasive fungal disease”, “fungal diagnostics”, “antifungal resistance”, “AFST”, “Candida”, “Aspergillus”, “Cryptococcus”, “diagnostic platforms”, “lateral flow assay”, “PCR”, “NAAT”, “MALDI-TOF”, among others. The focus of the search was on performance data of profiled diagnostic assays or platforms derived from independent studies that have been published in peer-reviewed literature, unless noted otherwise. Peer-reviewed articles, systematic reviews, and meta-analyses published in English were included, whereas editorials, commentaries, non-English publications, and studies lacking diagnostic performance data were excluded.

Expert consultations were conducted with WHO staff, members, and observers of the WHO Expert Group on diagnostics for fungal infections via targeted discussions and a virtual meeting held on May 16, 2024. The purpose of the expert consultations was validation of findings, identification of diagnostic gaps, and formulation of research and development priorities. In addition, discussions or semi-structured interviews were conducted with known diagnostic developers and manufacturers, wherein additional information was gathered or clarifications were made with respect to what is publicly available.

Estimates from 2019–21 suggest more than 6·5 million IFD cases and 3·8 million deaths occur annually, figures exceeding earlier projections dating from as early as 2009.^4^^,^^8^ Limited surveillance and poor diagnostic access obscure the true burden of IFDs, with the greatest effect in low-income and middle-income countries (LMICs), where delayed or missed diagnosis drives poor outcomes.^9^

AFR, driven by antifungal agent use in medicine and agriculture and environmental pressures, compounds this burden. For example, azole resistance in *Aspergillus* species is mainly linked to agricultural azole use, whereas echinocandin-resistant isolates from *Candida* spp largely arise from clinical exposure. These trends threaten the already scarce antifungal options.^3^^,^^10^ AFR worsens outcomes for IFDs, which can have mortality rates of more than 30–90% even when susceptible to drugs;^4^ between 1 and 1·5 million deaths annually are attributed to AFR.^11^ Diagnostic access is uneven, particularly in LMICs, but also in some high-income settings, leaving many IFDs undiagnosed. Whenever fungal infection is suspected but unconfirmed, empirical antifungal treatment is common, increasing costs, toxicity, and resistance.^2^ Delayed diagnosis of IFDs is associated with poor prognosis.^1^ The high morbidity and mortality of IFDs and increasing prevalence of AFR underscore the urgent need for accurate and accessible diagnostics that detect and identify the pathogen and define susceptibility or resistance profiles.^3^^,^^10^

Despite the urgent need, fungal diagnostics have lagged behind the development of tools for bacterial priority infections,^12^^,^^13^ owing to under-recognition of the threat and low commercial investment, which constrains research and development (R&D). In 2022, WHO published the fungal priority pathogens list, identifying 19 pathogens of critical, high, or medium priority (figure).^2^ In 2024, WHO analysed the antifungal clinical and preclinical pipelines^14^ and conducted a diagnostic landscape analysis to evaluate available and emerging tools and define R&D priorities.^15^ In this Review, we summarised those findings, identified diagnostic gaps, and outlined WHO’s R&D priorities to improve access to diagnostics globally, with a focus on LMICs.

**Figure:**
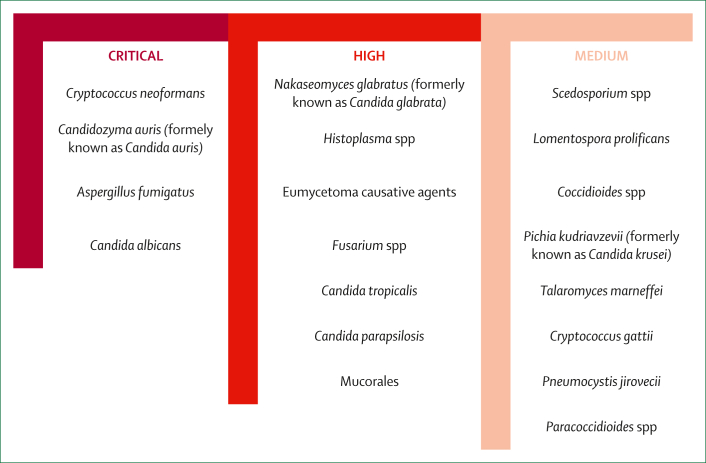
WHO fungal priority pathogens list The WHO fungal priority pathogens list^2^ is a catalogue of 19 drug-resistant fungi divided into three categories: critical, high, and medium priority. The list is the first global effort to systematically prioritise fungal pathogens, considering their unmet research and development needs and perceived public health importance.

## Findings of the first diagnostic landscape analysis for IFDs

In-vitro diagnostics for fungal pathogens encompass a range of methods. Classic phenotypic techniques, such as culture, microscopy, and histopathology, are primarily used for pathogen detection and, in some cases, identification, which could be enhanced by molecular or biochemical testing of cultured isolates. Non-culture-based approaches include immunological or biomarker assays that detect host immune responses (eg, antibodies) or fungal antigens and molecular assays (eg, nucleic acid amplification tests [NAATs]) that detect the presence of fungal DNA or RNA. These methods are often used in combination (eg, culture followed by biochemical testing or NAAT followed by sequencing) to achieve definitive identification. Once the pathogen is identified, antifungal susceptibility testing (AFST) assesses growth in the presence of antifungal agents, whereas AFR testing detects specific resistance mechanisms. The diagnostic methods are detailed in the following sections.

### Classic phenotypic methods for detecting fungal pathogens

Classic methods for the detection of fungal pathogens are direct culture, microscopy, and histopathology on clinical specimens. When testing sterile or deep sites, or both, these phenotypic methods can be specific and have been the foundation of the diagnosis of proven fungal infections since the early 20th century.

Fungal culture remains the gold standard for the diagnosis of IFDs, particularly when performed on sterile or deep clinical specimens, in which cases, isolation of the pathogen is considered definitive evidence of infection. The advantages of fungal culture include that it provides a broad range of detection; enables identification and strain characterisation through phenotypic or microscopic appearance, biochemical profiling, or molecular identification or typing; supports AFST; and is low cost relative to other diagnostic tests.

Nonetheless, fungal culture has several limitations, including variable sensitivity with more than 20–50% failure rates, wherein culture negativity cannot be used to exclude infection, which often makes empirical therapy necessary in individuals at high risk; delayed time to result, potentially exceeding 4 weeks, which limits the clinical utility in acute settings; the need for invasive specimens; and the need for appropriately trained personnel. In addition, not all fungi can be recovered through culture in routine laboratories (eg, *Pneumocystis jirovecii*).^1^^,^^10^^,^^16^, ^17^, ^18^

Furthermore, the recovery of fungi from non-sterile sites (eg, bronchoalveolar fluid) is not definitive of infection, as these organisms can represent colonisation, environmental contamination, or constituents of the normal microbiota. In such cases, clinical interpretation is essential to find out the significance of the detected organisms.

Microscopy can detect the presence of fungal elements in a clinical sample (preferably from infected body sites) within a few hours of sampling, but might not be sufficiently sensitive or specific when used alone.^19^^,^^20^ Similarly, histopathological examination of fungal cells in tissue samples is a rapid and generally cost-effective means of providing a presumptive diagnosis of IFDs while awaiting fungal culture results or in circumstances wherein no culture results are available or culture is no longer possible.^21^^,^^22^ However, both microscopy and histopathology require well trained technicians and histopathologists,^19^^,^^21^^,^^23^ and although the detection of fungal elements in a sterile or deep specimen is definitive of infection, genus-level or species-level identification is limited, highly subjective, and often impossible.^1^ As with culture, the presence of fungal elements in non-sterile samples requires clinical interpretation, but identifying the presence of morphological features such as hyphae, pseudohyphae, blastoconidia, or germ-tubes, is indicative of active fungal growth within the host.

Classic mycological methods are typically performed manually, but automated systems can standardise the processes and reduce the turnaround time for culture incubation, in addition to that for tissue staining, streaking, and inoculation of media for microscopic examination. However, all these systems are generally designed for use in high-throughput laboratories and are costly to implement, requiring appropriately trained technicians and suitable infrastructure to accommodate the instrumentation, including adequate space, consistent power supply, climate control, running water, access to transport, and reliable supply chains. These requirements restrict accessibility to the tests in LMICs, particularly in primary and secondary health-care facilities (table).^15^

**Table:** Description of the tiered laboratory system in low-income and middle-income countries

	Location in countries	Staff and role	Tests performed
Level 0	Community health posts and outreach	Health workers; no clinicians on site.	Lateral flow tests (immunochromatographic format or similar)
Level 1	Primary-care health-centre laboratories; mainly serve outpatients	Health workers; some clinicians on site. In the laboratory: microscopist.	Lateral flow assays and some smears by light microscopy (when a microscopist is available)
Level 2	Secondary care—district laboratories or hospitals	In the laboratory: technicians and assistants. In the clinic: physicians and other clinicians such as nurses commonly available on site.	All tests at level 1 plus: automated equipment for tests such as staining and blood culture, and instrument-based NAAT-based testing on small instruments designed for use near the point of care
Level 3	Tertiary care—regional or provincial laboratories and referral hospitals	In the laboratory: specialists or senior technicians. In the hospital: physicians and nurses.	All tests at level 1 or 2 plus: blood culture, full chemistry, identification, and AFST for first-line drugs; immunological testing and qualitative or quantitative NAAT-based testing, particularly on smaller instruments
Level 4	National and multicountry or large regional reference laboratories	In the laboratory: senior health specialists.	Blood culture, susceptibility testing and molecular testing, such as NAATs, drug resistance studies, and AFST for first-line and second-line medicines. Sequencing might be available.

Laboratory facilities in low-income and middle-income countries are usually characterised as a tiered network, level 0 (community or outreach facilities), level 1 (primary care), level 2 (district or secondary care), level 3 (regional or provincial or tertiary care), and level 4 (national and multicountry facilities). This network, the individuals served, and the testing services typically available at each level of the system are described herein.

NAAT=nucleic acid amplification test. AFST=antifungals susceptibility testing.

### Methods to identify fungal pathogens

Once a fungal pathogen has been recovered, several methods exist for identifying it. Phenotypic and microscopic appearance of the culture are often used to identify the genus, and in some cases, the species of fungi, but require extensive knowledge.^24^ Classic methods of identification can be supported by molecular and commercial biochemical tests (typically for yeasts) applied to culture and rely on the skills of trained personnel to perform the manual tests.^25^

Additional methods for the identification of invasive fungal pathogens include matrix-assisted laser desorption ionisation-time of light mass spectrometry (MALDI-TOF MS), attenuated total reflection-Fourier transform infrared (ATR-FTIR), hybridisation, and molecular methods.

MALDI-TOF MS is one of the most commonly used mass spectrometry methods for the identification of invasive fungal pathogens. The method ionises small, intact target pathogen proteins, generating a characteristic mass spectral profile that is compared to a large reference database of organism profiles for identification of pathogens from culture. Although primarily used for identification of yeasts (eg, *Candida* spp), MALDI-TOF MS has also shown improved results for identifying filamentous fungi (eg, *Aspergillus* spp), in addition to *Fusarium* spp, Mucorales, dimorphic fungi (eg, *Histoplasma capsulatum*, *Blastomyces dermatitidis*, *Coccidioides* spp, and *Talaromyces marneffei*).^17^^,^^25^ A major limitation in identifying fungi in the past was the reliance on incomplete or research-use-only fungal reference databases that could restrict species coverage, especially for moulds.^23^ However, manufacturers have now released comprehensive US Food and Drug Administration-approved and CE-marked spectral databases that include most clinically relevant filamentous fungi.

ATR-FTIR is a method for the detection and identification of pathogens (including fungi) that measures infrared absorption by passing infrared radiation through a sample, wherein some radiation is absorbed, resulting in a molecular vibration or fingerprint of the organism;^26^^,^^27^ the method requires the use of a spectral library. ATR-FTIR can be used to identify numerous fungal species, including *Aspergillus* spp, *Candida* spp, and *Fusarium* spp.

Several MALDI-TOF MS systems and one ATR-FTIR platform are commercially available. Each is highly automated and has a simple workflow with minimal hands-on time. Turnaround time is also fairly quick (less than 1 h to a few minutes following a positive culture), but their reliance on culture restricts utility. Moreover, due to the need for specially trained personnel and equipment requirements, these methods are generally limited to use at tertiary or higher health-care facilities in LMICs.

Hybridisation assays use labelled oligonucleotide probes to identify fungal pathogens, typically applied to a positive blood culture. The most commonly used hybridisation is peptide nucleic acid fluorescence in situ hybridisation (PNA-FISH), which uses probes targeting the ribosomal RNA of specific fungi, allowing visualisation under a fluorescence microscope.^17^^,^^25^ PNA-FISH assays are being phased out in favour of simpler and more universal PCR detection assays.

Molecular methods can identify fungal pathogens from positive cultures. To provide a broad range of identification, NAAT-based testing, in particular PCR, can provide species-specific or genus-specific identification through the use of targeted probes, or be combined with sequencing of the subsequent gene targets (DNA barcoding) to detect a broader range of possible pathogens.^3^^,^^28^ Sequencing methods can also detect the diversity of fungi at the genome level, in addition to the changing genetic evolution of fungi that cannot be detected by molecular testing, but can delay results.^29^

### Immunological methods for the detection of invasive fungal pathogens

Laboratory-based immunoassays for fungal diagnostics include both serological and biomarker-based approaches. Serological tests detect the host antibodies generated in response to exposure to the fungal pathogen; however, antibody presence does not necessarily indicate active infection. Common serological tests include antimannan antibody detection, *Aspergillus* IgG or IgE assays, and *Candida albicans* germ-tube antibody assays.

Biomarker assays detect fungal antigens or metabolites potentially released by the organisms during active growth or due to damage to the structural integrity of the fungal cell. These assays include β-(1-3)-D-glucan, galactomannan, and mannan antigen tests. Although biomarker positivity might reflect the presence of active fungal metabolites, it is not always indicative of disease and needs to be interpreted in the clinical context.^30^

Dependent on demand and accessibility, these immunoassays are fast, non-culture-based methods of detecting fungal priority pathogens in non-invasive specimens (eg, urine or saliva), minimally invasive specimens (eg, blood or serum), and invasive specimens (eg, cerebrospinal fluid and bronchoalveolar fluid).^1^^,^^23^^,^^31^ These immunoassays are primarily used to detect *Candida* spp, *Aspergillus* spp*,* and dimorphic fungi (eg, *Histoplasma* spp and *Coccidioides* spp) in individuals with compromised immunity, although their performance varies.^17^ Antibody tests might have reduced sensitivity in individuals with suppressed immunity, leading to false-negative results,^1^^,^^17^ whereas serum *Aspergillus* galactomannan assays perform well in individuals with neutropenia.^32^ Although commercially available immunoassays exist in each of these categories, automated assays require strong laboratory infrastructure, whereas manual assays require well trained laboratorians, either of which can limit access in low-resource settings.

Relative to laboratory-based assays, lateral flow immunoassays (LFIAs) are cost-effective, require minimal training, and offer rapid turnaround time; their use complements existing testing methods. The simplicity of LFIAs makes them suitable for point-of-care use across all health-care levels, including primary care facilities without laboratory infrastructure. However, some LFIAs require basic equipment or readers for interpretation, making them near-patient tests rather than point-of-care tests, and typically, their use should be complemented by an additional diagnostic investigation.

Commercial LFIAs are available for the detection of a narrow range of invasive fungal pathogens, including *Aspergillus* spp, *H* *capsulatum*, and *Cryptococcus* spp. Among these, cryptococcal antigen LFIAs show high sensitivity and specificity and are widely used, including in low-resource settings.^1^^,^^33^ However, the diagnostic performance of LFIAs for other invasive fungal pathogens varies. Factors such as an individual’s immune status, assay specificity, and cross-reactivity with other fungal species can affect accuracy.^23^ LFIAs for additional pathogens, including *P* *jirovecii*, *Fusarium* spp, Mucorales, and *Talaromyces* spp, are in development but still lack robust clinical validation.

Although the use of LFIAs can be conveniently implemented at lower levels of the health-care system, the management of IFDs and indeed many of the underlying conditions that predispose individuals to IFDs require patient management at higher levels of the health-care system. LFIAs can serve as a valuable rapid diagnostic screening tool to identify individuals for diagnostic investigation and management at higher levels of the health-care system.

### Molecular methods for the detection of invasive fungal pathogens

Molecular testing methods that detect specific nucleic acid sequences are widely used in clinical diagnostics. NAATs, particularly PCR-based assays, play an increasingly important role in detecting invasive fungal pathogens and are the most commonly used.^24^^,^^25^ PCR has been extensively evaluated for the detection of *Candida* spp, *Aspergillus* spp, *P* *jirovecii*, and Mucorales.^34^, ^35^, ^36^, ^37^, ^38^

Although fungal NAATs are less numerous and comprehensive, as compared with those for bacterial or viral pathogens, a growing number of commercial assays are available for both integrated and non-integrated PCR platforms.^13^^,^^15^ Quantitative or real-time PCR (rtPCR) allows rapid, sensitive detection and quantification, with added capabilities for species-level identification and AFR marker detection through fluorescent probes or high-resolution melt analysis.

Molecular-based test methods generally use samples directly from sterile sites (eg, blood, serum or plasma, or cerebrospinal fluid) or non-sterile sites (eg, bronchoalveolar fluid), which enables the detection of DNA and identification of fungal pathogens with a short turnaround time and good sensitivity and specificity. Some of these tests are also used to accelerate the identification of fungal pathogens in positive blood cultures.^3^^,^^17^^,^^23^^,^^25^^,^^33^^,^^39^ Most molecular tests can detect either *Candida* spp or *Aspergillus* spp, many can detect and quantify *P* *jirovecii*, and some can detect varying species of Mucorales. In addition, some commercially available molecular platforms can be multiplexed. Of these, blood culture-based multiplex panels are the most widely used.^3^^,^^9^ Multiplex panels directly testing cerebrospinal fluid include those for *Cryptococcus* spp, whereas panels testing bronchoalveolar fluid include those for some fungi (eg, *P* *jirovecii*), but are primarily research use only.^3^^,^^9^^,^^15^ Dimorphic fungi (eg, *Histoplasma* spp*, Coccidioides* spp, and *Talaromyces* spp) are not generally included in these panels.^9^ Consequently, most multiplex molecular panels target only a narrow range of fungal species, which can be improved through the use of sequencing, depending on assay design and when species-specific identification is needed.^38^

Molecular test methods typically provide faster results than phenotypic methods. Testing is best used in sophisticated laboratory settings requiring substantial infrastructure, which is not widely available in LMICs and other low-resource settings; however, emerging technologies such as isothermal amplification methods (eg, loop-mediated isothermal amplification, nucleic acid sequence-based amplification), CRISPR-based diagnostics, and biosensors show promise as low-cost, field-deployable tools, particularly in LMICs, although they require further clinical validation.^40^ Further, in the absence of a PCR assay with the capacity to detect and differentiate multiple potential fungal pathogens, the use of a PCR-specific pathogen requires at least a hypothesis that the fungus targeted by the assay is most likely causing the disease in the individual.^39^^,^^41^

### Phenotypic methods of AFST

Given the ability of fungal pathogens to develop resistance to antifungal medicines, AFST should be performed after pathogen identification, especially if the individual is at high risk of IFD, the organism is cultured from a sterile or deep site, antifungal therapy is being initiated or modified, or a combination of these.^42^ Although routine for bacterial infections and an important tool for managing IFDs, AFST is less accessible and lacks clinical breakpoints for many genera or species, but is increasingly important due to rising AFR.^24^^,^^42^ Phenotypic AFST evaluates fungal susceptibility by exposing fungi to antifungal agents in vitro and measuring the minimum inhibitory concentration (MIC) or minimum effective concentration (MEC) that halts growth.^24^^,^^43^ MIC or MEC values are interpreted on the basis of the species tested, using established clinical breakpoints to categorise susceptibility.^24^^,^^44^, ^45^, ^46^ Although the Clinical and Laboratory Standards Institute and the European Committee on Antimicrobial Susceptibility Testing have developed AFST standards, including MIC or MEC methods, clinical breakpoints, and epidemiological cutoff values that differ from species to species, breakpoints and cutoff values are available for only a limited number of species–antifungal combinations. With respect to fungal pathogens, the subject of MIC or MEC values, clinical breakpoints, and interpretive criteria are complex and beyond the scope of this Review.^42^^,^^44^, ^45^, ^46^

Three phenotypic AFST methods have been standardised by the European Committee on Antimicrobial Susceptibility Testing or the Clinical and Laboratory Standards Institute: broth microdilution (which is considered the reference standard), disc diffusion, and azole agar screening for *Aspergillus fumigatus*; additional methods include gradient diffusion and colorimetric MIC, which are considered alternative or complementary approaches, but are not standardised.^42^ Commercial semiautomated or automated systems for each of these methods are available.^15^ Although generally effective, these methods are reliant on culture, are costly, and require specialised, well trained microbiologists.^42^ Few interpretative criteria and a turnaround time of 24 h to 7 days make these methods impractical for many clinical laboratories, with most testing performed in reference or specialised facilities, which creates further delays, thus restricting the test’s clinical utility.^42^

Newer, faster AFST methods include imaging-based approaches such as the Cellometer Spectrum (Revvity, Waltham, MA, USA), microcolony imaging for *Candida* spp, and SensiFONG (in development, Linksium, Grenoble, France) for analyses of fungal cell walls. Non-imaging methods include voriconazole, itraconazole, and posaconazole plates for agar screening of azole resistance in *A* *fumigatus*. Additional methods, such as MALDI-TOF MS, flow cytometry, and isothermal microcalorimetry are promising, but not standardised or clinically available.^42^^,^^46^^,^^47^

### Molecular AFR testing

NAAT-based techniques, the most widely accepted methods for genotypic identification of fungal priority pathogens, can also assess AFR directly from clinical specimens without culture. Despite their extensive use in bacterial resistance testing, these techniques remain underutilised for AFR testing, and their utility is limited by the expanding range of genetic mechanisms underpinning AFR and a paucity of well curated repository databases of AFR mutations.^10^

A few commercial molecular assays integrate both fungal and AFR detection, mainly targeting mutations in *A* *fumigatus* and *P* *jirovecii*. However, the presence of mutations does not always correlate with increased MIC or MEC values. Although tests exist for identifying inherently resistant yeasts such as *Pichia kudriavzevii* (formerly known as *Candida krusei*) and *Candidozyma auris* (also known as *Candida auris*), no commercial assays detect AFR mutations in yeasts, highlighting a crucial gap.^10^^,^^48^ In addition, AFR databases similar to those available for antibacterial resistance are needed, to allow for comprehensive diagnostic coverage of existing AFR-associated mutations.^49^

### Commercial pipeline diagnostics and diagnostic advancements

Only few phenotypic or other diagnostic systems exist in the pipeline to detect and identify invasive fungal pathogens; assays in development are primarily molecular diagnostics using PCR-based methods. These assays include a chronic lung disease panel in development, which is expected to detect and identify *A* *fumigatus*, *C* *albicans*, and *Scedosporium* spp.^15^ In addition, a pan-Mucorales rtPCR assay and a multiplex rtPCR assay are in development for the detection and identification of some clinically significant Mucorales species.^15^^,^^50^

The pipeline of diagnostics that integrates identification and AFST or AFR testing is even more limited, with only two such systems found, one of which is a sepsis test panel in development that is expected to detect and identify a wide range of bacterial pathogens and smaller range of fungal pathogens (*Candida* spp*,* including *C* *auris* and *Cryptococcus gattii* [formerly known as *Cryptococcus neoformans* var *gattii*]), in addition to performing AFR testing.^15^

The use of next-generation sequencing (NGS), including metagenomic NGS and whole-genome sequencing (WGS), is becoming a more appealing option in clinical laboratories because it can provide broad-range projection, high throughput, and faster results compared with slower conventional methods such as fungal culture, histopathology, microscopy, serological assays, and phenotypic AFST, complementing syndromic testing directly from clinical samples.^28^ NGS can be applied to a cultured fungal organism providing fungal identification, with WGS also providing phylogenetic and epidemiological information, and potentially, information on AFR.^28^

The application of NGS to provide syndromic diagnostic approaches represents a key development across microbiology. However, the adoption of NGS for fungal disease is progressing more slowly than that for other infections, even with syndromic diagnostic approaches, in which fungi are often under-represented.^41^ At this stage, the platforms are primarily being used in clinical microbiology laboratories within hospitals and similar settings for research use only. NGS platforms have drawbacks for use in low-resource settings, including high equipment cost and computer infrastructure, need for specialised expertise in bioinformatics, and inadequate standardisation of methods and workflow, among others.^28^^,^^41^

## Discussion

In summary, classic phenotypic methods for detection of fungal pathogens, such as culture, microscopy, and histopathology, remain foundational in the diagnosis of fungal infections caused by priority pathogens. However, these approaches present substantial limitations, particularly in LMICs. The approaches rely heavily on culture, which is slow and labour intensive, especially when performed manually, and require substantial laboratory infrastructure and specialised personnel. As a result, the use of these approaches is largely confined to tertiary care centres.

For instance, culture and identification of *Candida* species can take 2 to 5 days, whereas identification of moulds can require up to 4 weeks, delaying the initiation of appropriate therapy and driving empirical approaches.^1^^,^^19^^,^^39^ Sample viability or detection of the pathogen can also be compromised during transport, particularly when specimens need to be referred to centralised laboratories. Moreover, both culture and microscopy might have low sensitivity, and the operational demands of these methods often exceed the capabilities of many health-care facilities in LMICs.

Classic methods of fungal pathogen detection and identification can be supported by molecular and commercial biochemical tests, primarily for yeasts,^25^ applied to culture. Additional test methods, such as MALDI-TOF MS, provide faster identification following culture growth and have the potential to provide applications beyond identification, such as AFST.^51^ Molecular methods, such as PNA-FISH, PCR amplification, and sequencing, can also be used for identification from culture. With the exception of PNA-FISH, which is being phased out in favour of PCR, these methods require specialised infrastructure, equipment, and expertise, typically restricting their use to high-resource settings.

Non-culture-based methods of pathogen detection, such as immunoassays and biomarker-based serodiagnostics, offer more rapid detection of fungal pathogens than conventional culture, microscopy, and histology but often lack genus-level or species-level resolution. These methods still require laboratory infrastructure, specialised reagents, and trained personnel, thereby restricting their broad applicability in low-resource settings. LFIAs provide a more accessible, point-of-care alternative. However, with the exception of the cryptococcal antigen LFIA, the diagnostic performance of LFIAs remains inconsistent. Consequently, LFIAs are best suited as initial screening tools, with confirmatory testing required for definitive diagnosis.

Molecular methods, in particular NAAT-based tests, such as PCR, play an important role in the detection of invasive fungal pathogens, allowing rapid and high-throughput testing. However, these tests remain impractical for most LMIC facilities due to infrastructure requirements, and are therefore typically located in reference facilities or health-care settings caring for substantial populations of individuals at high risk. Furthermore, commercial platforms typically detect a limited number of fungal species, and if AFR testing is incorporated, then only a few genetic mechanisms are targeted, necessitating additional procedures (eg, sequencing), which restrict test utility.

Despite the increasing importance of AFR, commercial AFST methods are slow, costly, and not widely available or standardised across all fungal species on the fungal priority pathogens list. The need for culture and appropriately trained personnel are additional challenges. Similarly, only a few molecular systems combine fungal pathogen identification and AFR testing. These tests have not been widely used, have limited species coverage, and, along with the need for appropriate training and infrastructure, also require AFR databases.

### Diagnostic gaps and needs

Given that a definitive (ie, proven) diagnosis of an IFD is rare and that, clinically, individuals are often managed with probable, possible, or unclassified IFDs,^1^ this assessment of available diagnostics to detect and identify fungal priority pathogens and to perform AFST or AFR testing highlights key gaps in tests or testing platforms at all levels of health systems, particularly in LMICs (panel 1).

Panel 1 **Identified gaps in diagnostics for IFDs in low-resource settings**

• Very limited ability to perform fungal culture for detection or identification and antifungal susceptibility testing below tertiary care facilities in low-income and middle-income countries (LMICs); difficult to perform microscopy or histopathology in secondary care facilities due to the need for specially trained personnel.^52^, ^53^, ^54^
• Few automated or semiautomated tests are commercially available for identification of yeasts and almost none for filamentous fungi.^25^^,^^55^ Most tests have inadequate sensitivity or specificity.
• Limited availability of non-culture-reliant diagnostic methods, apart from those for the most commonly occurring pathogens on the WHO fungal priority pathogens list (eg, *Candida* spp, *Aspergillus fumigatus*, *Cryptococcus neoformans*, and *Pneumocystis jirovecii*), and lack of accessibility to these tests, except at tertiary health facilities and higher in LMICs.
• Scarcity of multiplex tests or platforms, in particular, tests suitable for use in secondary health settings, to detect and identify fungal pathogens directly from clinical specimens (no culture required) with antifungal susceptibility testing or antifungal resistance testing done on a separate platform or integrated on the same platform.
• Few lateral flow immunoassays with sufficiently strong performance to detect and identify individual invasive fungal pathogens, and no available multiplex lateral flow immunoassays for use at any level of the health system, including primary care.

The main premise of this Review is that diagnosing IFDs will be facilitated by tests that can detect and identify antifungal susceptibility or resistance for a wider range of fungal priority pathogens; are faster and easier to use; offer greater accuracy; and are more affordable than currently available tests. These diagnostics should ideally be accessible at all levels of the health system and should include tests appropriate for use at primary and secondary health facilities in LMICs and other low-resource settings, recognising that testing capabilities in these facilities will vary by country, infrastructure, training, supply chain capabilities, and other factors, and that individuals tested will typically require clinical management and confirmatory testing at higher levels of health care.

Additional key characteristics of diagnostics for fungal priority pathogens include those that detect and identify pathogens when used during the early stages of an infection; have good sensitivity and specificity; discriminate among species or genera; can quantify fungi as a guide to distinguish between colonisation and disease when interpreted as part of a comprehensive clinical investigation; and detect AFR.^9^^,^^21^ No single diagnostic platform exists that meets all these criteria.^21^

## R&D priorities

WHO has published a set of R&D priorities for IFD diagnostics in LMICs and other low-resource settings over the next 3–5 years categorised by settings for which the tests are most appropriate and in order of test complexity (lowest to highest; panel 2).^15^

Panel 2 **Research and development priorities based on landscape gap analysis**

**Primary health-care facilities and higher**

• Lateral flow immunoassays with improved performance for use with whole blood, serum, or other sample matrices (eg, urine, swab samples, upper respiratory tract samples) to detect and identify a broader range of invasive fungal pathogens than those currently available.

Suggested action: Develop a consensus-driven target product profile (TPP) for lateral flow immunoassays, possibly for simultaneous detection and identification of more than one fungal pathogen, with good sensitivity and specificity, suitable for use at all levels of health systems in low-income and middle-income countries (LMICs), with recognition that these tests are designed for rapid triage or screening and should not be used in isolation for final diagnosis at any level of the health-care system.

**Secondary health-care facilities and higher**

• Simplified blood culture platform to expand blood culture usage to secondary laboratories with easier to use systems or adaptations to existing systems, to make them more accessible.

Suggested action: Build on existing simplified blood culture TPP,^56^ and ongoing work by intergovernmental organisations and other public health organisations, to support the development of systems for, and the implementation and health systems integration of, newer culture methods appropriate for secondary facilities in LMICs.

• Simplified automated assays or systems for the detection or identification and antifungal susceptibility testing (AFST) of filamentous fungi, which are limited in number and are primarily available for yeasts.

Suggested action: Develop a consensus-driven TPP for automated assays or systems that can both identify and perform AFST on a wider range of invasive fungal pathogens than those currently available, to be performed on lower cost, simplified instrumentation. Set improved sensitivity or specificity requirements. Work on characterisation of resistance mutations that are clinically significant and build a comprehensive curated database that could serve for development of genotypic assays for resistance prediction.

• Simplified, sample-in, result-out diagnostic systems with the capability to detect and identify multiple WHO fungal priority pathogens (eg, *Candida* spp*, Cryptococcus, Aspergillus* spp*,* among others), on a single assay panel, and ideally, to perform AFST or antifungal resistance (AFR) testing on an integrated platform without culture.

Suggested action: Develop a consensus-driven TPP for a multiplex diagnostic platform suitable for use at secondary health-care facilities and higher in LMICs that can simultaneously detect or identify multiple fungal priority pathogens directly from whole blood or other sample matrices, and ideally, can perform AFST or AFR testing on an integrated platform.

## Conclusions

To improve detection and identification of fungal priority pathogens and the diagnosis of IFDs, there is a crucial need for simpler, faster, and more sensitive commercial diagnostic methods that can detect a broader range of fungi than those currently available and that do not require culture. There is also an acute need for faster, simpler methods of AFST and AFR testing than what is currently achievable with existing methods. In each case, testing methods adapted for use in LMICs and other low-resource settings are of particular importance. In addition, diagnosis of IFDs remains complex and multifaceted, with most diagnoses based on a sufficient probability of infection rather than a definitive diagnosis. The combined use of fungal diagnostics remains most likely for the near future, and strategies to accommodate combined diagnostics need to be identified and validated.

The WHO R&D priorities enumerated in panel 2 are intended to inform public health actors, including diagnostic developers, of the assays that are most needed for fungal priority pathogens and can be useful across multiple settings in LMICs and beyond. The development of the suggested target product profiles provides guidance with respect to key operating characteristics of the diagnostics, including target use, target use setting, performance required (eg, sensitivity and specificity), and more.

The road from discovery to delivery of in-vitro diagnostics is a complex process involving many steps. These steps include basic research and proof of concept; platform and prototype development; product optimisation; premarket validation; clinical trials; scale-up or manufacturing; and commercial product release. The process can take 5 years or more, even after prototype development, and can cost millions of US dollars. In parallel with R&D efforts, diagnostics capacity should be strengthened through a range of measures, including investing in diagnostic services, developing a skilled workforce, and taking policy measures to ensure equitable and timely access to diagnostics. Health system integration is important to facilitate uptake, reimbursement, and continued use of diagnostic testing.

Funding will be required to support diagnostic developers, for new in-vitro diagnostics to be successfully developed and implemented. In addition, support will be required from global stakeholders in various areas, including but not limited to further development of specimen banks; development of international standards, reference reagents, and reference panels for key diagnostic markers; strong coordinated efforts by stakeholders to streamline evaluations of new diagnostic platforms to speed regulatory approval; and targeted technical assistance on the adoption of new in-vitro diagnostics. WHO and other actors have many cross-cutting activities to support the development of such new in-vitro diagnostics, including those for fungal priority pathogens. Ongoing advocacy for R&D and timely access to these technologies are needed to address IFDs.

## Declaration of interests

MM and TR are consultants to the WHO AMR Division. TTB has provided consultancy or participated in the scientific advisory boards of Sefunda, CARB-X/Mod, BiosparQ, and the Global AMR R&D Hub. PJD has received research support from Avir Pharma and Oxoid; and honoraria for educational talks from bioMérieux, Diasorin, and Micronostyx. GG-E has received non-monetary support from IMMY. AA-I has received research support from Scynexis paid to her institution; honoraria for educational talks from Gilead, Pfizer, and Mundipharma; and has participated in the scientific advisory board of Basilea Pharmaceuticals. SC-AC has received untied research support from F2G. BWT works at CARB-X; has participated in the advisory boards for the Right Foundation, Nesta, and Pathways to Antimicrobial Clinical Efficacy (PACE); and has received grants for travel from Mérieux Foundation and Value-Dx via Innovative Medicines Initiative (IMI). PLW discloses research support from Bruker in 2022, IMMY in 2022, and non-monetary support from Virclia and Fast MDx. All other authors declare no competing interests.
